# Staged hybrid repair for refractory type B aortic dissection with persistent false lumen perfusion: Integration of thoracic endovascular aortic repair, frozen elephant trunk, and laser fenestration

**DOI:** 10.1016/j.xjtc.2026.102448

**Published:** 2026-05-28

**Authors:** Sivaraj Pillai Govindasamy, Marco Lizwan, Azamal Ibrahim G. Mulla, Deborah Harrington

**Affiliations:** a Department of Cardiothoracic Surgery, National Heart Centre Singapore, Singapore; b Department of Cardiothoracic Surgery, Liverpool Heart and Chest Hospital, Liverpool, United Kingdom

**Figure:**
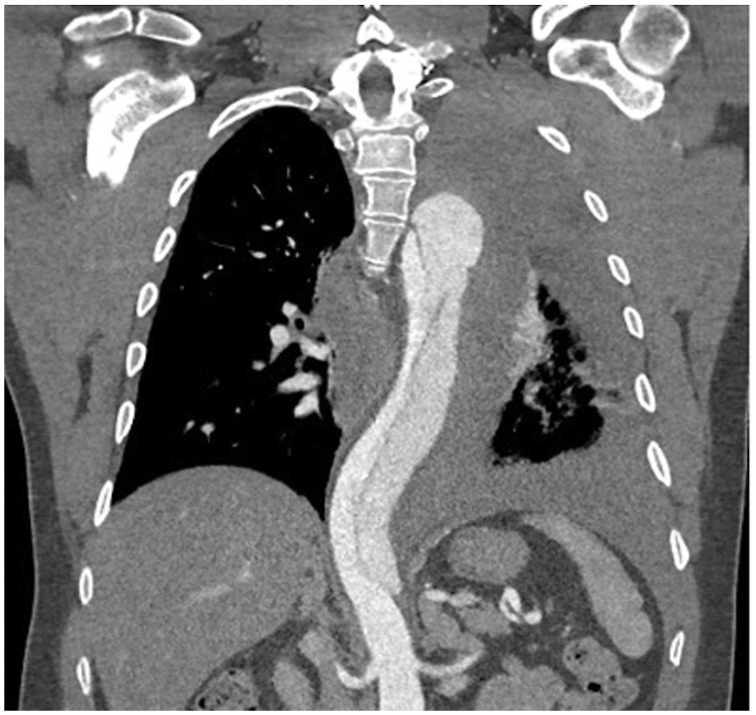
CT aortography showing complex type B aortic dissection.

**Central Message**

Persistent false lumen perfusion after TEVAR in complicated type B aortic dissection may require staged hybrid intervention to achieve durable aortic remodeling.

Acute type B aortic dissection (TBAD) is a complex condition, with outcomes closely linked to complications such as rupture, malperfusion, or persistent symptoms. Thoracic endovascular aortic repair (TEVAR) is the preferred first-line therapy in complicated cases, because it promotes false lumen thrombosis through the sealing of the primary entry tear. However, incomplete false lumen exclusion remains a recognized limitation and is associated with adverse aortic remodeling and an increased likelihood of reintervention.^1^

In selected patients, persistent perfusion of the false lumen necessitates escalation beyond conventional TEVAR. Hybrid strategies incorporating open surgical repair and adjunctive endovascular techniques have emerged as viable solutions in such scenarios.^2^ We describe a case of refractory TBAD requiring a staged hybrid approach integrating TEVAR, frozen elephant trunk (FET), and advanced endovascular techniques including laser fenestration. Although hybrid to TBAD has been described, there is limited literature detailing a staged escalation strategy in the setting of persistent false lumen despite the use of both TEVAR and FET. This report highlights a pragmatic, stepwise approach culminating in adjunctive endovascular techniques to achieve definitive aortic remodeling.

## Case Presentation

Written informed consent was obtained from patient for the publication of clinical data and images. Institutional review board approval was not required.

**Figure 1:**
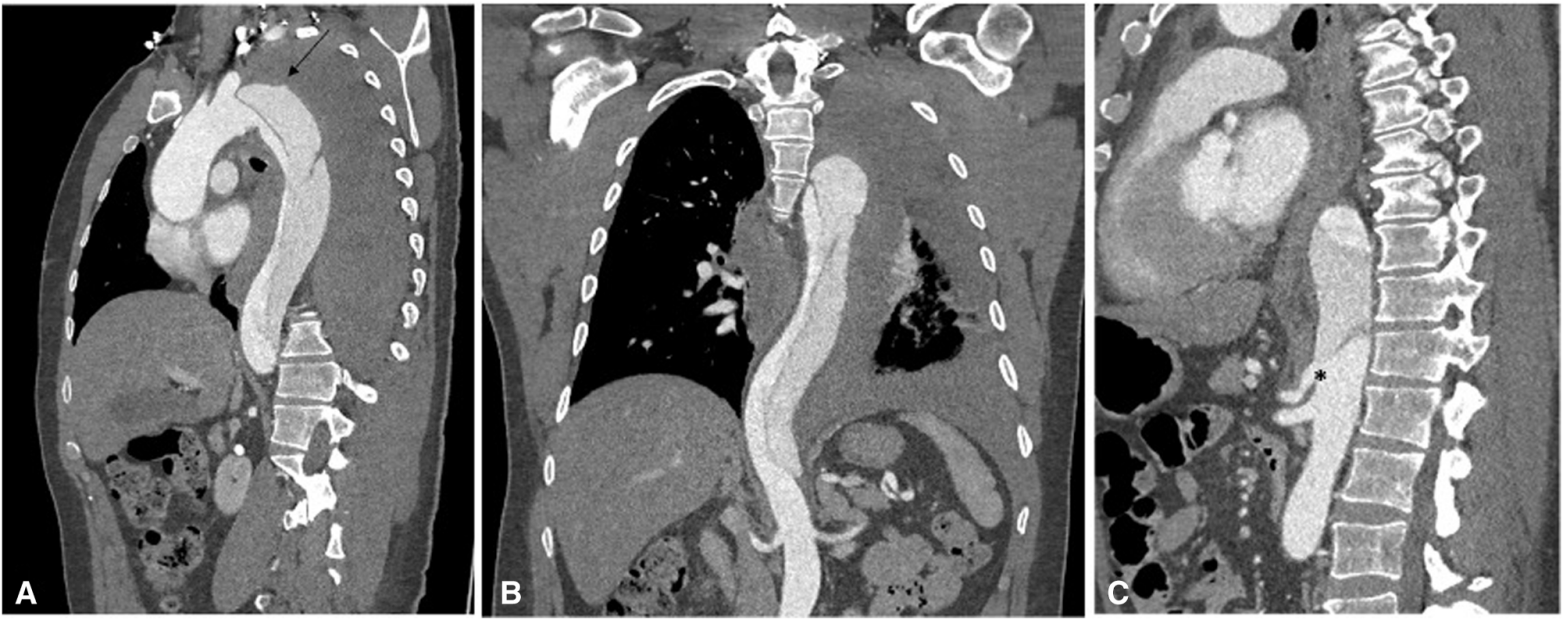
CT aortography in complex type B aortic dissection. Oblique sagittal CT reformat (A) shows complex dissection flap distal to the origin of the left subclavian artery, with the smaller true lumen pushed to the anteromedial aspect of the arch of aorta and descending thoracic aorta by the larger false lumen, which shows faint contrast opacification. Note the left hemothorax (attenuation 45 HU) and the irregular contour of the false lumen superiorly (*black arrow*), suggesting ruptured dissection. Coronal oblique (B) and sagittal oblique (C) CT reformats show the distal extent of the dissection, reaching just above the celiac axis origin (*asterisk*). *CT*, Coronary angiography.

A 46-year-old man with a history of hypertension presented with acute severe back pain and a syncopal episode. Computed tomography of aorta demonstrated a complicated TBAD with a large left-sided hemothorax and features suggestive of contained rupture (Figure 1). The dissection extended along the descending thoracic aorta with a patent false lumen. Given the presence of hemothorax and concern for ongoing bleeding, urgent intervention was undertaken.

**Figure 2:**
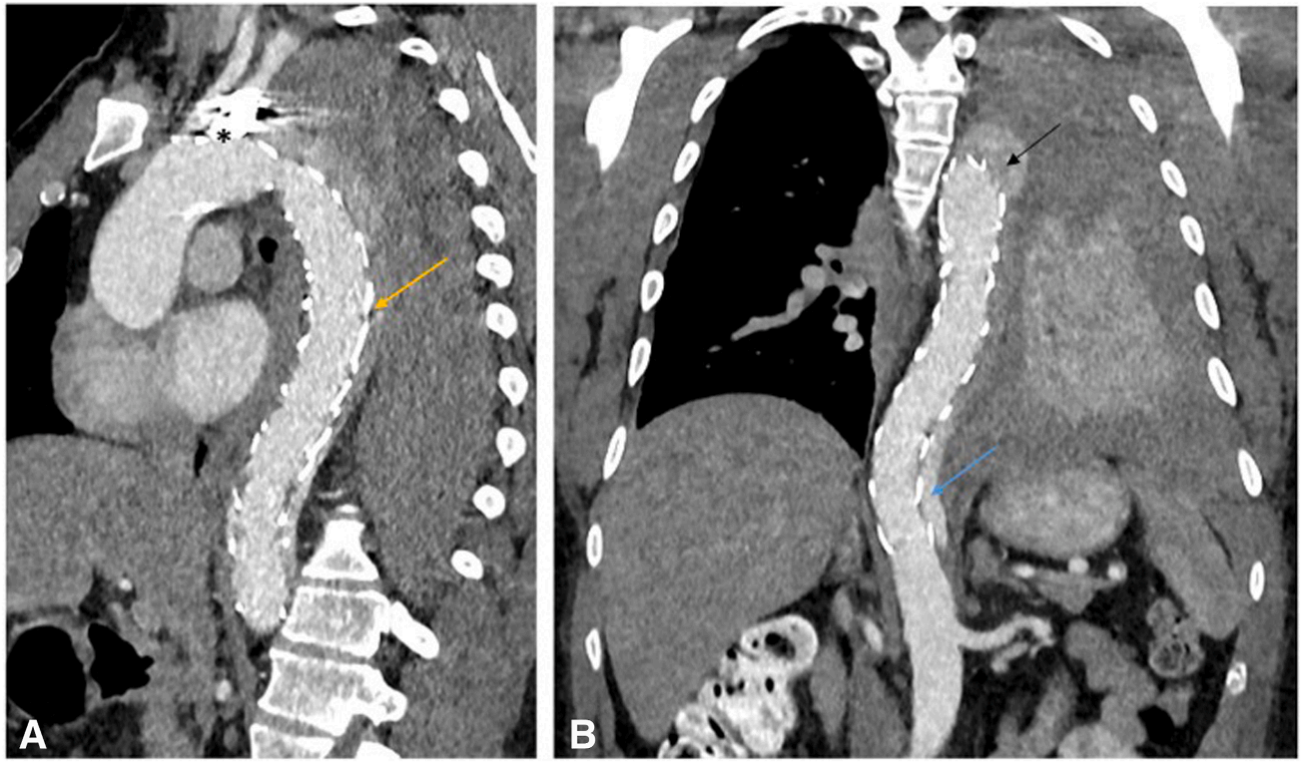
CT aortography after TEVAR. Sagittal oblique CT reformat (A) shows TEVAR stent in situ (*yellow arrow*) and the coiled left subclavian artery (*asterisk*). There is faint contrast opacification of the false lumen, which is better appreciated on the coronal oblique reformat (B), at the level of distal descending thoracic aorta (*blue arrow*) and distal arch (*black arrow*) with thrombosed false lumen in between, suspicious for endoleaks at proximal and distal ends of the stent. Residual hemothorax with atelectasis of the left lung is also noted. *CT*, Coronary angiography; *TEVAR*, thoracic endovascular aortic repair*.*

An initial TEVAR was performed via femoral access on the day of admission. TEVAR was selected as first-line therapy in accordance with current practice for complicated TBAD, allowing rapid control of the proximal entry tear with lower procedural morbidity. A thoracic stent graft was deployed to cover the proximal entry tear, with intentional coverage of the left subclavian artery to achieve an adequate proximal seal. The stent graft was sized with approximately 10 to 15% oversizing relative to the proximal landing zone. Coverage extended to the mid-descending thoracic aorta. The left subclavian artery origin was embolized using detachable coils to prevent retrograde perfusion. Angiography performed at completion confirmed satisfactory positioning of the stent graft; however, persistent false lumen perfusion was noted, likely attributable distal re-entry and no classical type I or III endoleak was identified (Figure 2).

In view of these findings and the high-risk presentation, a multidisciplinary decision was made to proceed with early surgical escalation as the result of persistent false lumen perfusion. On the second day of admission, the patient underwent total arch replacement with FET. This strategy was used to provide a more durable proximal seal and facilitate downstream aortic remodeling by extending coverage into the descending thoracic aorta.^3^

**Figure 3:**
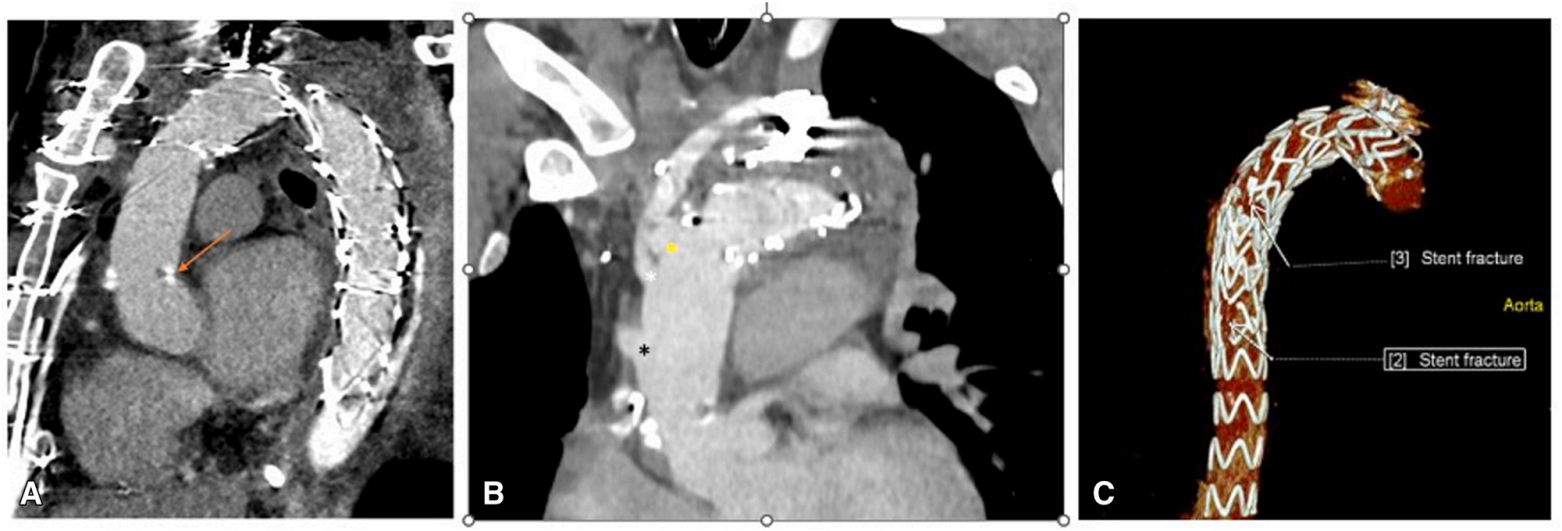
CT aortography after ascending aorta and total arch replacement with frozen elephant trunk surgery. Sagittal oblique CT reformat (A) depicts the proximal anastomosis site of ascending aorta (*orange yellow*), without any para-anastomotic leak. Note the persistent endoleak posterior to the TEVAR distally. The endoleak seen previously at the level of the arch is not visualised. The reimplanted brachiocephalic, left common carotid and left subclavian arterial grafts (indicated by *black, white and yellow asterisks*, respectively) are well opacified as seen on the coronal oblique reformat image (B). 3D reconstruction image (C) shows stent fracture at two sites (*arrows*) in the TEVAR stent. *CT*, Coronary angiography; *TEVAR*, thoracic endovascular aortic repair*.*

The early postoperative course was initially stable. However, by day 14, the patient developed progressive respiratory compromise, and imaging demonstrated complete opacification of the left hemithorax. Repeat computed tomography revealed persistence of the false lumen without overt contrast extravasation (Figure 3). Video-assisted thoracoscopic evacuation of the hemothorax revealed no active bleeding identified, suggesting ongoing low-pressure perfusion from the false lumen.

**Figure 4:**
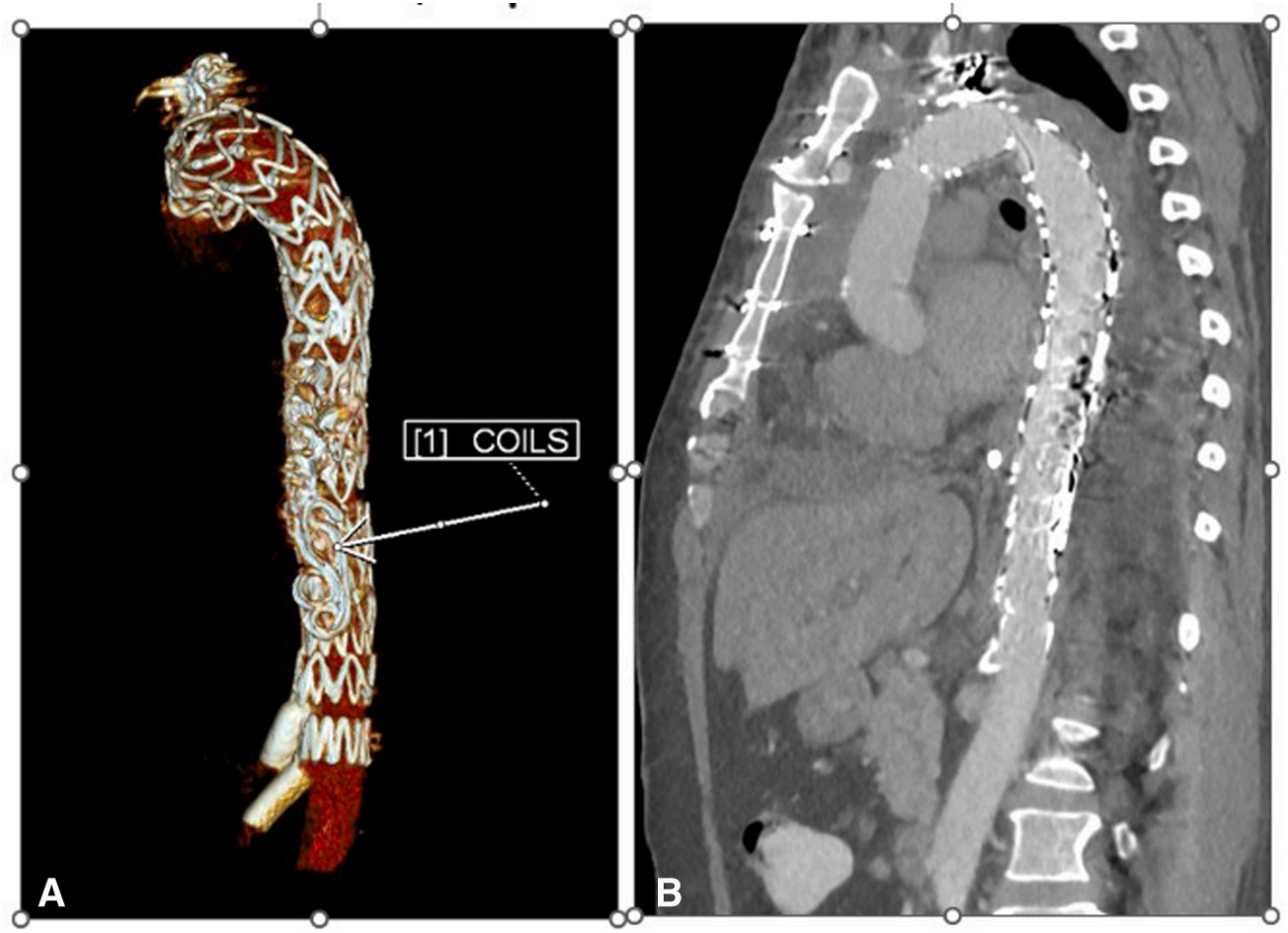
CT aortography post endoleak repair. 3D reconstructed image (A) shows the coils along the posterolateral aspect of the TEVAR stent used to occlude the endoleaks. There is extension of the stent inferiorly with stenting of the celiac axis and SMA as well. Sagittal oblique reformat (B) does not show any opacification of the false lumen, indicating successful management of the endoleaks. *CT*, Coronary angiography; *SMA*, superior mesenteric artery; *TEVAR*, thoracic endovascular aortic repair*.*

Given the persistence of both hemothorax and false lumen patency, a third-stage endovascular intervention was undertaken on day 21. The false lumen was selectively cannulated, allowing targeted coil embolization. A tapered dissection stent graft was then deployed to reinforce exclusion. To maintain visceral perfusion, in situ laser fenestration was performed, followed by stenting of the celiac artery. Partial compromise of the superior mesenteric artery during stent deployment necessitated placement of a balloon-expandable stent to ensure adequate flow. Completion angiography demonstrated successful exclusion of the false lumen with preserved visceral perfusion (Figure 4).

Follow-up imaging confirmed complete thrombosis of the false lumen and resolution of the hemothorax. The patient recovered steadily and was discharged on postoperative day 45. Serial imaging demonstrated durable aortic remodeling with no recurrence of false lumen perfusion.

Genetic testing was not performed during the index admission, because there were no clinical features suggestive of underlying connective tissue disease. This represents a limitation and may warrant consideration in future similar cases.

## Discussion

TEVAR has transformed the management of complicated TBAD; however, persistent false lumen perfusion remains a clinically significant challenge. Data from the International Registry of Acute Aortic Dissection highlight that incomplete aortic remodeling is associated with poorer long-term outcomes and increased need for reintervention.^1^

The FET technique provides an effective adjunct in cases in which the proximal seal is inadequate or disease extends proximally. By combining open surgical repair with endovascular stenting, FET facilitates more extensive exclusion of the false lumen and promotes favorable remodeling.^3^ It is conceivable that FET may create a pressurized blind segment in the presence of distal re-entry tears. However, in this case, persistent false lumen perfusion was more likely driven by distal re-entry rather than isolated proximal pressurization. Alternative strategies may have included extended endovascular coverage or delayed intervention, although the presence of hemothorax necessitated active treatment.

Adjunctive endovascular techniques such as false lumen embolization and in situ fenestration have emerged as valuable tools in addressing these limitations. In situ laser fenestration represents an off-label modification of commercially available devices and should be undertaken with caution. However, it remains a valuable adjunct in complex anatomy in which preservation of branch vessel perfusion is critical.^4^

This case illustrates the importance of a staged and adaptive treatment strategy. Management was guided by ongoing clinical and radiological assessment. Such an approach aligns with contemporary evidence supporting individualized and multidisciplinary care in TBAD.^5^

## Conclusions

Persistent false lumen perfusion after TEVAR in complicated TBAD may necessitate staged hybrid intervention. Integration of surgical and advanced endovascular techniques, including laser fenestration, can achieve durable aortic remodeling and favorable clinical outcomes.

## Conflict of Interest Statement

The authors reported no conflicts of interest.

The *Journal* policy requires editors and reviewers to disclose conflicts of interest and to decline handling or reviewing manuscripts for which they may have a conflict of interest. The editors and reviewers of this article have no conflicts of interest.
